# Tuning SWCNT Length to Optimize the Rate–Efficiency–Stability Triad in Nanofluidic Water Channels

**DOI:** 10.3390/molecules30234548

**Published:** 2025-11-25

**Authors:** Shu-Peng Wang, Qi-Lin Zhang, Zhi-Jun Ma, Ju-Xiang Li, Zhen-Yan Lu, Rong-Yao Yang

**Affiliations:** 1 Hunan Provincial Key Laboratory of Intelligent Sensors and Advanced Sensor Materials, School of Physics and Electronics, Hunan University of Science and Technology, Xiangtan 411201, China; 2 School of Mathematics-Physics and Finance, Anhui Polytechnic University, Wuhu 241000, China; 3 School of Science, Sun Yat-sen University, Shenzhen Campus, Shenzhen 518107, China

**Keywords:** single-walled carbon nanotubes, water transport, molecular dynamics simulation, water-chain bridging mechanism, nanofluidics

## Abstract

This work shows that the length of single-walled carbon nanotubes is critical in governing the trade-off among the rate, efficiency, and stability of pressure-driven water transport. A critical length of 1.06 nm marks the transition in the transport mechanism from a thermal-fluctuation-dominated regime to an ordered water-chain mode. This transition is driven by the evolution of the potential of mean force with tube length, which progresses from a flat landscape to a high-barrier profile and ultimately forms a low-resistance tunnel in long nanotubes. Notably, this tunnel endows the water chain with an enhanced ability to restore its continuity, allowing it to bridge fracture gaps as wide as 7 Å even in the absence of an external pressure difference. These insights reveal a length-dependent mechanism that could revolutionize CNT–hydrogel hybrids for biomedical applications.

## 1. Introduction

The pursuit of artificial nanochannels that rival the efficiency of biological systems is a central theme in modern nanotechnology, promising transformative advances in water purification [1,2,3], energy conversion [4,5,6], and medicine [7,8]. Among various platforms, single-walled carbon nanotubes (SWCNTs) are exemplary candidates, celebrated for their atomically smooth interiors that enable ultra-fast [9], nearly frictionless water transport [9,10], leading to novel physical phenomena, such as the formation of ordered, single-file water chains and even new phases of ice [11], and achieving transport rates that are orders of magnitude beyond classical predictions [9,12,13]. In reality, defects are inevitable arising from fabrication and operation [14,15,16]. These structural imperfections are known to dramatically alter transport behavior, often inducing anomalous diffusion [17] and rendering a purely rate-focused optimization strategy obsolete. This highlights an urgent need to shift the design paradigm from maximizing sheer speed to balancing speed, efficiency, and transport stability. In fact, maintaining the continuity of the water chain in the presence of defects is a highly challenging and controversial topic. For example, previous studies have explored in detail the stretchability limits of nanoscale water bridges and highlighted the significant difficulties they face regarding stability [18].

This study directly addresses this pressing challenge by investigating how a simple geometric parameter, the length of the SWCNT, critically mediates this complex trade-off. By incorporating precisely defined structural fractures into SWCNTs in molecular dynamics (MD) simulations, we reveal a powerful, length-dependent design principle. We demonstrate that short SWCNTs exhibit high initial flow but suffer from inefficiency and rapid failure due to chaotic bidirectional backflow and the sensitivity of water chains to structural fractures; in contrast, longer SWCNTs achieve enhanced stability and efficiency despite a reduced maximum rate. They sacrifice a fraction of the maximum rate to achieve near-perfect unidirectional efficiency and, most importantly, exhibit an exceptional ability to maintain continuity in the water chain, dramatically allowing it to autonomously bridge and sustain transport across surprisingly large fracture gaps (up to 7 Å) even in the absence of a pressure difference. Our findings pivot the role of length from a simple rate modulator to a sophisticated design parameter for achieving optimal system-level resilience. In particular, in the biomedical field, by leveraging this length-dependent water–chain bridging mechanism, CNT-hydrogel hybrid materials are poised to thoroughly revolutionize multiple applications. For example, in targeted drug delivery for cancer treatment, the material can maintain structural integrity under physiological stresses while achieving controlled release of therapeutic drugs [19]; in tissue engineering scaffolds for neural or cardiac repair, it promotes cell migration and regeneration through enhanced electrical conductivity and resilience [20,21,22]; and in terms of advanced wound dressings, it combines antibacterial properties with robust transport continuity even in the presence of micro-fractures, significantly accelerating the healing process [23]. This integration not only enhances the biocompatibility of the hybrid materials but also opens new avenues for personalized medicine, underscoring the transformative potential of this discovery in addressing real-world health challenges.

## 2. Methods

**Figure 1:**
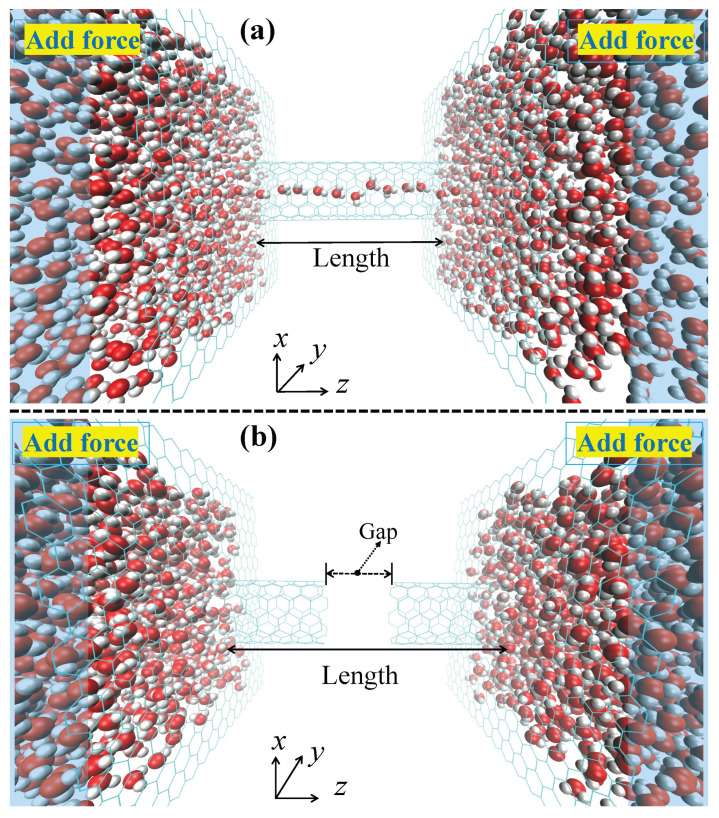
Schematic of the simulation system. The model consists of a (6,6) armchair single-walled carbon nanotube (SWCNT) embedded between two parallel graphene membranes and submerged in a water box with a fixed $3.5\times 3.5{\mathrm{nm}}^{2}$ cross section and containing a fixed 1802 water molecules. The box length in the *z*-direction ($L_{z}$) is variable and was determined for each system via NPT equilibration (see Section 2), ensuring $L_{z}$ scales proportionally with the nanotube length *L*. The blue regions indicate where an external force is applied to induce directed flow. (**a**) The system with an intact SWCNT of a specified length. (**b**) The model for a fractured nanotube, where the SWCNT is separated into two segments by a defined Gap.

As shown in Figure 1, the system included an uncapped (6,6) armchair SWCNT embedded between two parallel graphene membranes along the *z*-direction. To accommodate the nanotube, a circular pore was created at the center of each graphene sheet by removing carbon atoms. The radius of this pore was set to be the radius of the (6,6) SWCNT plus an additional 3 Å, ensuring no covalent bonds between the tube and the membrane. The system was centered at the coordinate origin. The ends of the nanotube were positioned to be co-planar with their respective graphene membranes (i.e., the distance between the tube end and the membrane plane is 0 Å). To simulate a fracture, the nanotube was modeled as two disjoint SWCNT segments separated by a gap of a specified distance. Their total length was equal to that of the intact nanotube before fracturing. The carbon atoms at the edges of the fracture were kept fixed in their positions throughout the simulation, and the dangling bonds of these edge carbon atoms were not passivated. MD simulations were conducted using NAMD3 with a 1 fs time step. All bonds involving hydrogen atoms were constrained using the SHAKE algorithm. A constant temperature of 300 K was maintained by Langevin dynamics (damping coefficient of 1 ps^−1^) applied to all atoms. Periodic boundary conditions were applied in all directions; this ensures that water molecules exiting the downstream reservoir are reintroduced upstream, preventing any artificial density gradients or back pressure effects. Electrostatic interactions were managed using the particle mesh Ewald method. For non-bonded van der Waals and short-range electrostatic interactions, a real-space cutoff of 1.2 nm (12 Å) was used, with a potential switching function applied starting at 1.0 nm (10 Å) to ensure smooth truncation; no analytical long-range tail corrections were employed. The simulation was based on the CHARMM force field and TIP3P water model. To achieve directed flow, an external force of 1.345 pN was applied to water molecules within 15 Å of both ends in a water box containing a total of 1802 water molecules.

**Figure 2:**
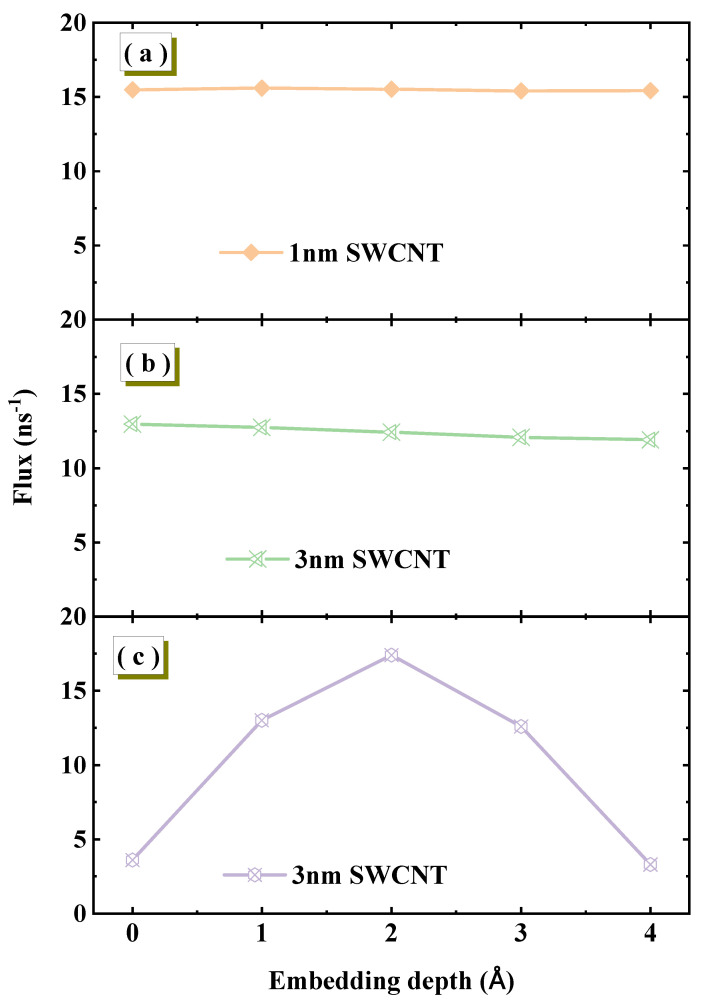
The effect of SWCNT embedding depth within the graphene membrane on water flux under different conditions. (**a**,**b**) Fully equilibrated NPT systems with SWCNT lengths of 1.34 nm and 3 nm, respectively. (**c**) A non-equilibrated system in which the SWCNT length is 3 nm.

To ensure that the core conclusions of our study are not influenced by specific model geometric parameters, we first conducted a critical simulation aimed at investigating the sensitivity of water flux to the embedding depth of SWCNTs in graphene membranes. We designed three distinct systems. First, two systems fully equilibrated in the NPT ensemble were examined. For an SWCNT with a length of 1.34 nm, varying the embedding depth within the 0–4 Å range shows no significant dependence of water flux, which remains stable at approximately 16 ns^−1^, as shown in Figure 2a. Increasing the nanotube length to 3 nm yields a similar result, as shown in Figure 2b. This indicates that under equilibrium conditions, nanotube length is not the primary factor controlling this sensitivity. To further validate the importance of equilibrium, we constructed a comparative system using the same 3 nm SWCNT but with insufficient equilibration in the NPT ensemble, and the results are shown in Figure 2c. Under this non-equilibrium condition, flux exhibits high sensitivity to embedding depth, fluctuating dramatically with small depth changes. Therefore, whether the system is fully equilibrated is the decisive factor determining flux stability. Under equilibrium conditions, flux remains stable against embedding depth variations, while non-equilibrium systems display strong, irregular dependence. This sensitivity is directly related to the equilibrium state of the system. For systems that have been sufficiently equilibrated in the NPT ensemble, achieving physically realistic water densities, the water flux exhibits insensitivity to smaller changes in embedding depth. However, in systems that have not been sufficiently equilibrated, the water flux shows a strong, non-physical dependence on the embedding depth. Based on this conclusion, all simulations used for analysis in our study start from rigorously equilibrated states.

To quantify the water transport, two key metrics are employed. The flux is defined as the net number of water molecules translocating through the nanotube per unit time, calculated as $(N_{\mathrm{forward}}-N_{\mathrm{backward}})/\Delta t$, where $N_{\mathrm{forward}}$ and $N_{\mathrm{backward}}$ are the total counts of forward and backward translocation events, respectively, and Δt is the total simulation time. The flow is defined as the total number of translocation events in both directions per unit time, given by $(N_{\mathrm{forward}}+N_{\mathrm{backward}})/\Delta t$. With these definitions, both flow and flux share the unit of ns^−1^. Consequently, the unidirectional transmission efficiency, η, is a dimensionless ratio defined as η=Flux/Flow.

A critical aspect of our methodology is the system equilibration, which addresses the determination of the simulation box size. All systems were constructed with a fixed number of 1802 water molecules. Before data collection, each system (corresponding to a specific nanotube length, *L*) was rigorously equilibrated in the NPT ensemble (at 300 K and 1.01325 bar, using the Langevin piston method with a piston period of 200 fs and a decay time of 100 fs) until key properties, such as system temperature, density (approaching 1.0 g/cm^3^), and total energy, reached a stable plateau (see Figures S1–S3 in the Supplementary Materials for representative equilibration plots).

This NPT equilibration procedure allows the simulation box length in the *z*-direction ($L_{z}$) to dynamically adjust for each specific nanotube. Consequently, as the nanotube length (*L*) increases, the final box length ($L_{z}$) also increases proportionally. This robust approach ensures that the water reservoirs at both ends of the nanotube remain sufficient and consistent across all systems, thereby avoiding artificial finite-size effects from the simulation box.

Following this NPT equilibration, the system was switched to the NVT ensemble (300 K, using Langevin dynamics) for a 45 ns production run for data analysis. To ensure statistical reliability, this entire process was repeated four times for each case using different initial velocities, which were generated from a Maxwell–Boltzmann distribution at 300 K to ensure statistical independence of the trajectories. The results presented are the mean values from these independent runs, and the error bars represent the standard error of the mean.

To analyze the structural integrity of the water chain, a hydrogen bond is considered to exist if the oxygen–oxygen distance is less than 3.5 Å and the H-O⋯O angle is less than or equal to 30°. These geometric criteria are standard and have been widely adopted in molecular dynamics studies of water in nanoconfined systems [24,25].

The potential of mean force (PMF) for water molecules along the nanotube axis (*z*) was calculated using the Boltzmann inversion method, according to the relation $PMF\left(z\right)=-k_{B}T\ln\left(\rho \left(z\right)/\rho_{0}\right)$. The density profile, ρ(z), was obtained by taking the average over four independent, unbiased MD simulation trajectories. Each trajectory consisted of 45,000 frames, providing robust sampling of the system. In the calculation, $k_{B}$ is the Boltzmann constant, *T* is the temperature (300 K), the nanotube axis was divided into bins of 0.5 Å width, and $\rho_{0}$ is the bulk water number density (0.0334 molecules/Å^3^). To quantitatively characterize the PMF profiles, we performed a Fast Fourier Transform (FFT) analysis. After subtracting the mean value of each PMF curve to remove the DC component, we calculated the Power Spectral Density (PSD). To ensure a fair comparison of the intrinsic landscape features across different nanotube lengths, the analysis was grounded in Parseval’s theorem, which relates the total energy variance in the spatial domain to the integrated power in the frequency domain. The total power ($P_{\mathrm{total}}$), defined as the integral of the PSD over all wave numbers, reflects the overall roughness of the energy landscape. The peak power ($P_{\mathrm{peak}}$) corresponds to the power of the dominant periodic component and is proportional to the square of the primary energy barrier’s amplitude. The resulting unit of power from this analysis is (kcal/mol)^2^.

## 3. Results and Discussion

**Figure 3:**
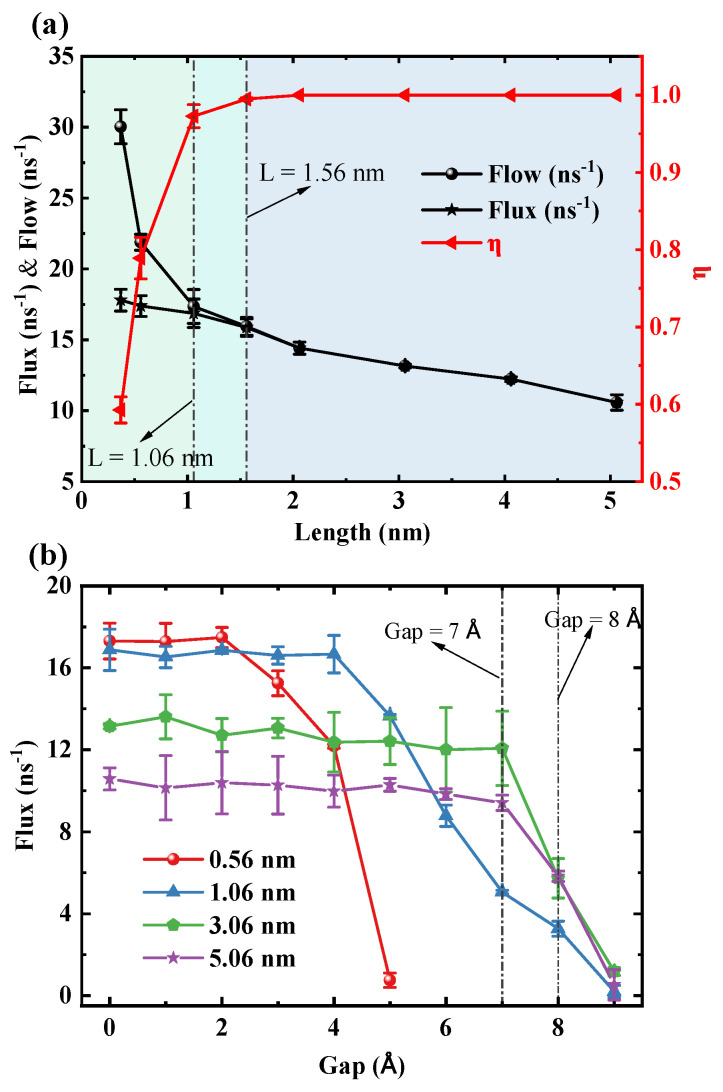
(**a**) The response of three key physical quantities to the variation in the length of SWCNTs. The black curve with circle markers, the black curve with star markers, and the red curve with triangle markers represent flow, flux, and unidirectional transmission efficiency (η), respectively. (**b**) The variation of flux with the fracture gap when SWCNTs are fractured into two segments for different tube lengths. The red, blue, green, and purple curves correspond to tube lengths of 0.56 nm, 1.06 nm, 3.06 nm, and 5.06 nm.

Figure 3a illustrates a significant trade-off between transmission rates (flow and flux) and unidirectional transmission efficiency (η=Flux/Flow). Specifically, both flow and flux decrease with increasing length of the SWCNT, with the most pronounced reduction in flow occurring in short tubes (L<1.06 nm). Conversely, η increases rapidly with tube length, reaching 0.97 at 1.06 nm and approaching 1 in longer tubes, thereby achieving nearly perfect unidirectional transport. Thus, 1.06 nm represents a critical length at which the system maximizes transmission efficiency while sacrificing some transmission rate. To confirm that this transition is not an artifact of the TIP3P model, we performed comparative simulations using the SPC/E and TIP4P/2005 water models. These tests confirmed that the critical transition in transport efficiency at approximately 1.05 nm is a robust phenomenon, consistent across all three models (see Figures S4 and S5 in the Supplementary Materials). Figure 3b reveals that the ability of the water chain within the SWCNT to bridge the gap strongly depends on length. Driven by pressure, for short SWCNTs of 0.56 nm and 1.06 nm, the flux declines sharply at gaps of 3 Å and 5 Å, respectively. The gap value that causes the sharp decline in flux is defined as the critical gap. As the length increases to 3.06 nm and 5.06 nm, the system exhibits enhanced stability, with the critical gap delayed until 8 Å. Notably, beyond a length of 3 nm, the critical gap ceases to increase with further length extension, indicating a saturation effect. We also validated the robustness of this water-chain bridging mechanism. While the quantitative value of the critical gap is, as expected, model-dependent (5 Å for SPC/E and 3 Å for TIP4P/2005 compared to 7 Å for TIP3P), the fundamental physical phenomenon of a critical breakdown gap was validated in all cases (see Figure S6 in the Supplementary Materials). This confirms that the mechanism is robust, although its precise range is modulated by the specific water–water interaction potential.

**Figure 4:**
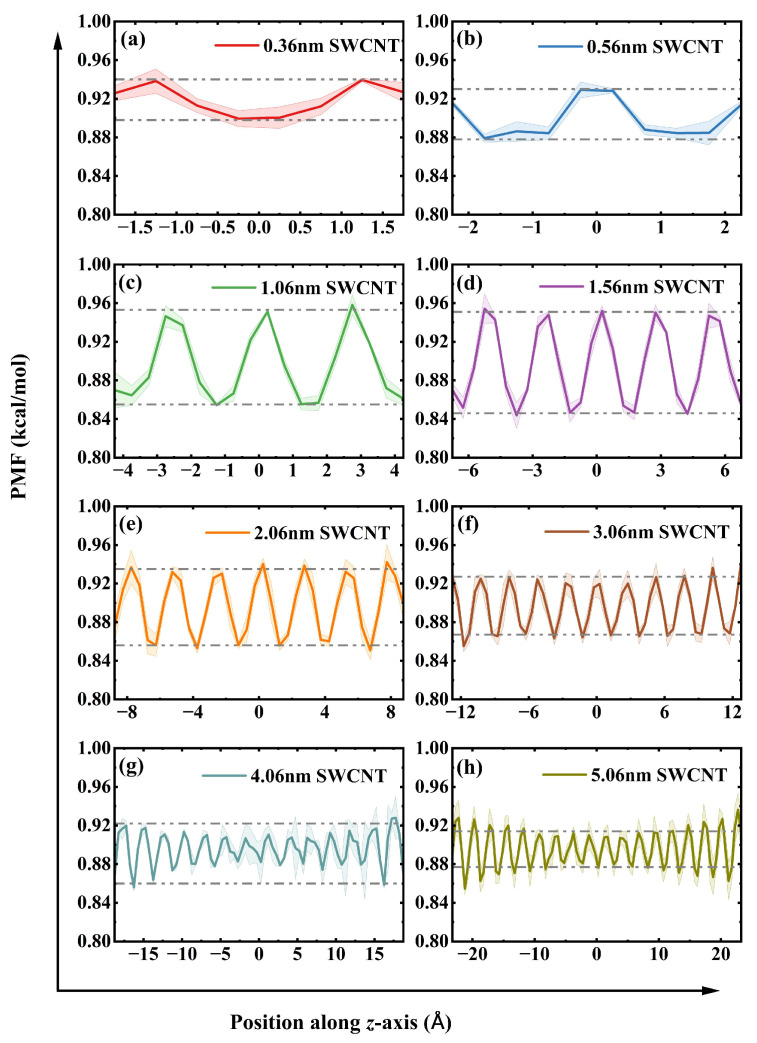
Potential of mean force (PMF) curves for water molecules inside the tube with different lengths: (**a**) 0.36 nm, (**b**) 0.56 nm, (**c**) 1.06 nm, (**d**) 1.56 nm, (**e**) 2.06 nm, (**f**) 3.06 nm, (**g**) 4.06 nm, and (**h**) 5.06 nm. These profiles reveal the evolution of the transport mechanism, transitioning from a shallow well in short tubes (L<1.06nm) to an ordered locking state in intermediate-length tubes (L=1.06∼1.56nm) and ultimately to a stable energy tunnel in long tubes (L>1.56nm). The shaded regions represent the standard deviation.

**Figure 5:**
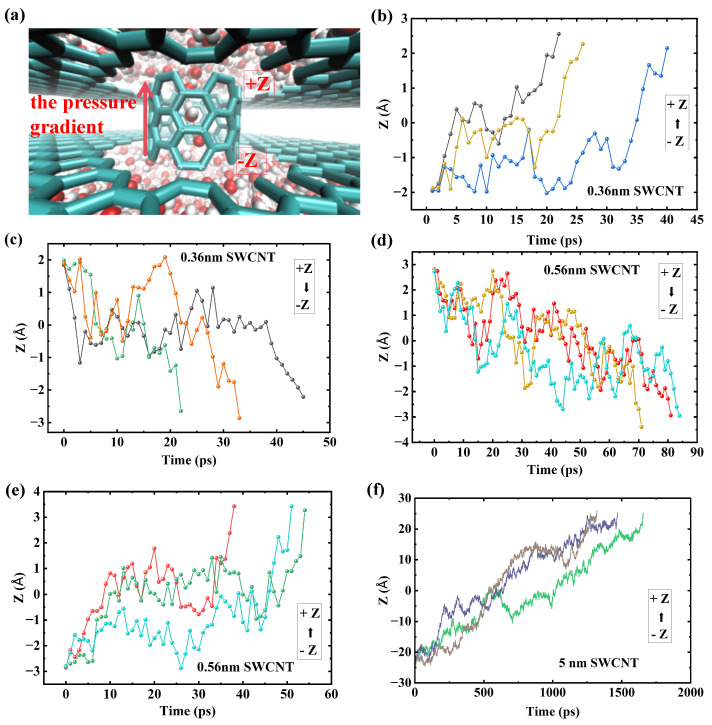
Trajectories of water molecules inside SWCNTs under a pressure gradient. The colored lines represent the trajectories of individual water molecules. The x-axis represents time, and the y-axis represents the position inside the nanotube. (**a**) A snapshot illustrating the single-file water chain within the nanotube. Trajectories for the short (0.36 nm) SWCNT show motion (**b**) with the pressure gradient (from −Z to +Z) and (**c**) against the gradient. Similarly, for the 0.56 nm SWCNT, trajectories show motion (**d**) with and (**e**) against the gradient. (**f**) Trajectories in a long (5.06 nm) SWCNT, demonstrating predominantly unidirectional flow.

The trade-off between water transport rate, efficiency, and stability as a function of tube length can be explained by analyzing the potential of mean force (PMF) along the tube axis for water molecules. The PMF elucidates the effective free energy landscape experienced by water molecules within the nanoconfined channel, directly determining their dynamic behavior. We first confirm that all PMF curves exhibit thermodynamic symmetry along the nanotube axis (*z*-direction). This symmetry is a direct consequence of the identical nature of the water reservoirs at both ends of the uncapped SWCNT, confirming that the channel itself possesses no inherent energetic bias for directional transport. As illustrated in Figure 4, the PMF can be categorized into three stages. The first stage corresponds to a disordered and perturbation-sensitive shallow well. In short SWCNTs (L<1.06nm), the PMF curve is characterized by a flat profile with minimal energy barriers, indicating strong coupling between the water inside the tube and the reservoirs at both ends. Consequently, random thermal fluctuations at the tube’s openings are sufficient to reverse the effect of the externally applied pressure difference, thereby triggering significant backflow. This intense bidirectional motion results in extremely low η yet paradoxically yields a high flow due to the inclusion of numerous forward and backward movements. The low barrier landscape is too easily overcome by thermal noise, resulting in poor net efficiency. Consistent with this theoretical framework, we observe bidirectional, intermittent burst-like motion of water molecules inside the tube, aligning with previous research findings [26]. This clear transition between distinct energy landscapes (Figure 4) and their corresponding transport behaviors strongly supports the concept of multi-state diffusive mobility, where distinct fast and slow transport states co-exist in narrow nanochannels [27]. To illustrate this behavior, Figure 5 shows the trajectories of water molecules in SWCNTs of varying lengths under a pressure gradient. In short tubes (0.36 nm and 0.56 nm), the motion is characterized by bidirectional, burst-like jumps, with water molecules moving both along (from −Z to +Z) and against (from +Z to −Z) the pressure gradient. This reflects the dominance of thermal fluctuations, leading to low unidirectional efficiency. In contrast, for longer tubes (5.06 nm), the trajectories show primarily forward motion with occasional backward steps, but the overall trend is unidirectional, consistent with the formation of a stable, ordered water chain in the energy tunnel regime. This fluctuation-driven, burst-like motion observed in short tubes (Figure 5b–e) is characteristic of the anomalous diffusion regimes commonly found in such single-file systems [28]. Direct quantitative evidence for this ‘burst-like’ dynamic is provided in the Supplementary Information (see Figure S7), which plots the instantaneous total flow for the *L* = 0.56 nm system, showing its highly unstable and erratic magnitude.

**Figure 6:**
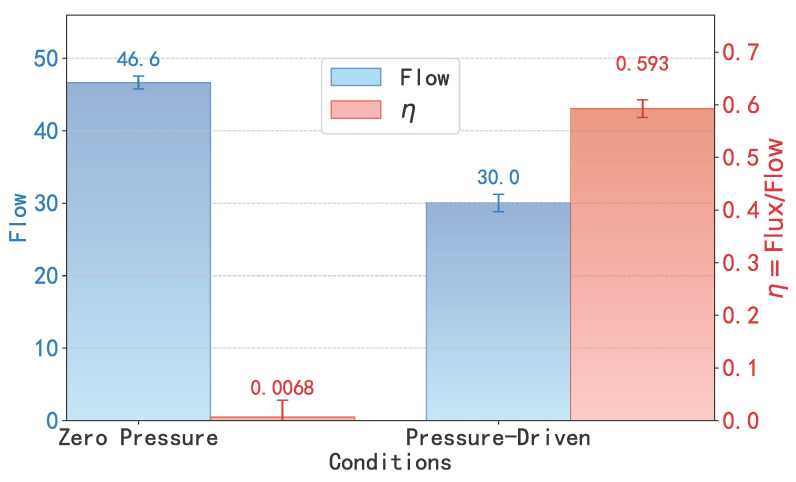
Comparison of the flow in short SWCNTs (L=0.36nm) under zero external pressure difference and pressure-driven conditions. The flow under zero pressure is higher, demonstrating the dominant role of thermal fluctuations in short tubes. Comparison of the flow (total throughput, left axis) and transmission efficiency (η, right axis) for short SWCNTs (L=0.36nm) under zero external pressure and pressure-driven conditions. This plot highlights a critical trade-off: the zero-pressure system shows a higher total flow, dominated by thermal fluctuations, but has a near-zero efficiency (η≈0). In contrast, the pressure-driven system sacrifices total flow but achieves a significant unidirectional efficiency (η≈0.59).

At the same time, this theoretical framework is further corroborated by direct comparison of flow under zero and pressure-driven conditions, as illustrated in Figure 6. For a short SWCNT (L=0.36nm, well below the critical length of 1.06nm), the flow under zero external pressure difference is notably higher than that under the applied pressure (1.345 pN). This counterintuitive behavior reveals that in the shallow-well regime, thermal fluctuations are the dominant driving mechanism of water transport. The complex, non-white (or ‘colored’) nature of this molecular-scale thermal noise, which is itself governed by the hydrogen bond network, is fundamental to understanding this regime [29]. Crucially, the figure also visualizes the associated trade-off in efficiency (η, right axis). Under zero pressure, this high total flow is driven by chaotic, bidirectional thermal motion, resulting in a net efficiency η that is nearly zero. Once an external pressure is applied, it partially suppresses these random fluctuations, which reduces the total flow but establishes a net directional transport, causing η to rise significantly (from ≈0 to ≈0.59). These results provide direct evidence that in short SWCNTs, transport is governed by thermal noise rather than by external forces, in full agreement with the low-barrier PMF profile and the low values of $P_{\mathrm{peak}}$ and $P_{\mathrm{total}}$ obtained from FFT analysis.

**Figure 7:**
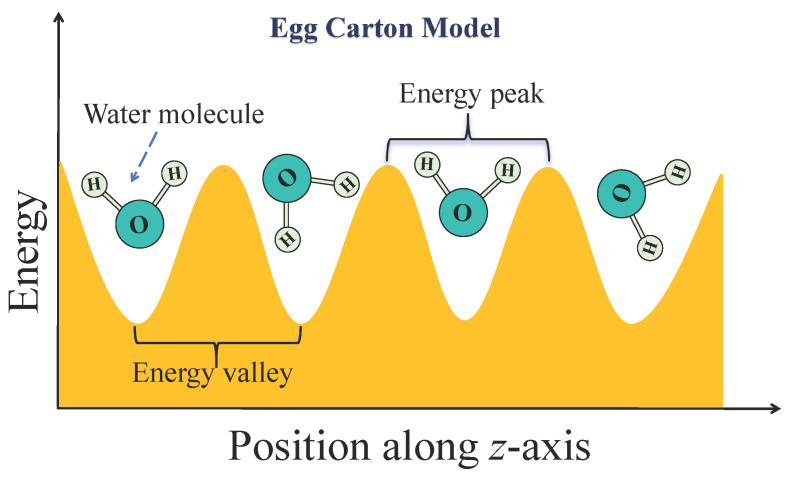
A schematic of the egg carton model. The *x*-axis represents the SWCNT axial position, and the *y*-axis represents energy. Troughs are low-energy valleys corresponding to stable residence sites for water molecules. Peaks are high-energy barriers.

The second stage is marked by an ordered locking phase with dramatically increased fluctuations. As the length extends to approximately 1.06 to 1.56 nm, the transport mechanism undergoes a fundamental transformation. Water molecules inside the tube organize into a structured, single-file water chain interconnected via a hydrogen bond network. This phenomenon, where interfacial confinement dictates the formation of non-bulk, ordered water structures at room temperature, is a fundamental effect observed not only in 1D channels but also as ordered bilayers on 2D surfaces [30]. Such strong ordering inherently constrains the molecular motion, significantly limiting the rotational freedom of the water molecules [31], a mechanism that has also been shown to govern other nanoscale phenomena like evaporation [32]. This ordering is directly reflected in the PMF, which exhibits high-amplitude periodic fluctuations throughout the tube [33]. This amplification of the energy barriers is a direct consequence of the water’s ordering; such ordered interfacial structures are known to possess an anomalously low dielectric constant, which reduces electrostatic shielding and thus magnifies the underlying potential landscape [34]. Each peak represents a free energy barrier that a water molecule must surmount to transition from one stable position to the next. For the entire water chain, this resembles being firmly locked within the tube by an egg carton-like energy landscape. To visualize this, Figure 7 presents a schematic of the egg carton model, where the axial position along the SWCNT corresponds to an alternating energy landscape of peaks and valleys. The troughs represent low-energy valleys, which are stable residence sites for water molecules, while the peaks are high-energy barriers that must be overcome for migration between stable positions. This model aids in understanding the PMF’s significant fluctuations with distinct amplitude and periodicity, corresponding to the energy differences between stable and transitional configurations of water molecules in the ordered locking phase. This rugged profile effectively suppresses backflow by requiring collective energy to surmount the barriers. Crucially, the energy provided by random, reverse thermal fluctuations at the tube openings is insufficient to collectively drive the water chain over this series of high internal energy barriers, thereby strongly suppressing the influence of thermal fluctuations and maximizing unidirectional efficiency (η). This is achieved despite the trade-off of slightly higher resistance to forward flow, which reduces the absolute rate compared to the shortest tubes.

**Figure 8:**
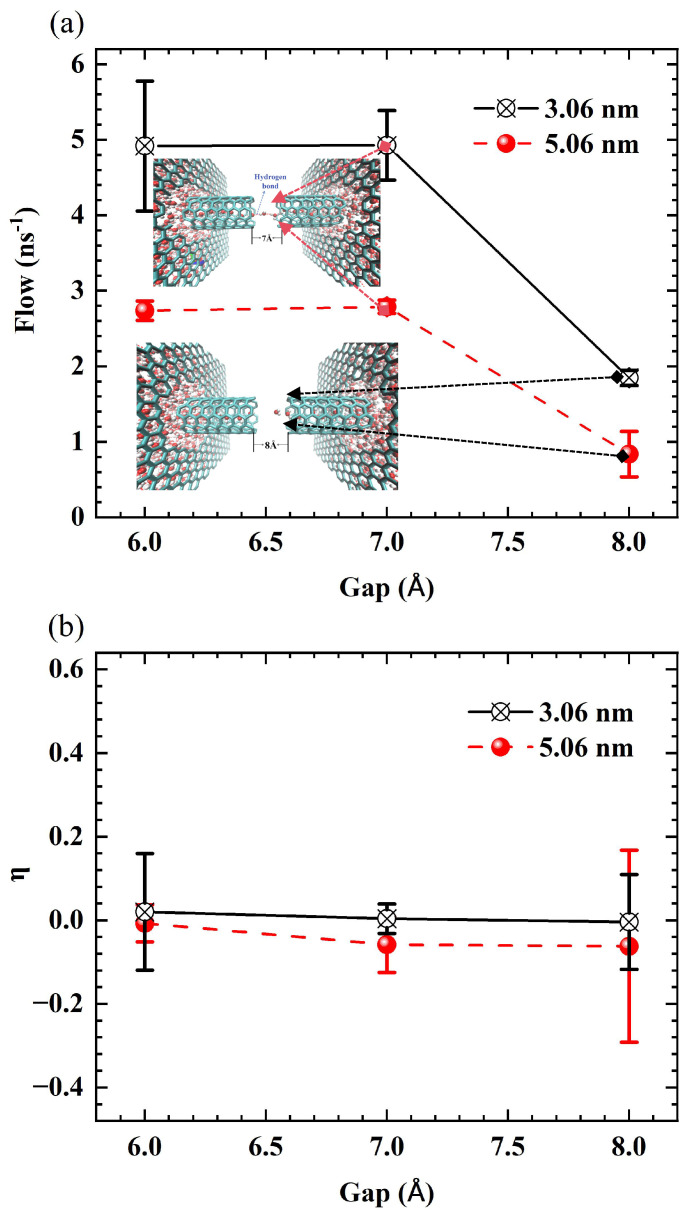
(**a**) Total flow and (**b**) unidirectional efficiency (η) as a function of gap for SWCNTs of lengths 3.06 nm (black line) and 5.06 nm (red line) under zero external pressure difference (ΔP=0).

The third stage occurs when the length exceeds 1.56 nm, gradually forming a stable tunnel transport mode, characterized by the transition of the water chain from a rigid short rod to a flexible long chain. These energy barriers govern the bridging capability of the fractured water chain. For short tubes of 0.56 nm, end effects hinder water molecules from bridging larger gaps. In nanotubes ranging from 1.06 nm to 1.56 nm, the PMF displays high-amplitude, drastic fluctuations across the entire domain. This implies that water molecules are confined in deep potential wells, and crossing a fracture requires overcoming a high energy cost. Consequently, it can only tolerate smaller fracture gaps. As the length increases to 3.06 nm, the overall fluctuation amplitude of the PMF decreases significantly, indicating that the energy barrier for the migration of water molecules is reduced, thereby empowering the water chain with astonishing bridging capabilities to autonomously span larger distances (up to 7 Å) in the absence of a pressure difference. In 5.06 nm tubes, the PMF adopts a high at both ends, low in the middle profile, with pronounced high-energy barrier regions near the entrance and exit due to end effects, while the extensive central region forms an energy tunnel with small fluctuation amplitude. This low-resistance characteristic is in excellent agreement with the mechanism that ordered water structures, such as monolayers, can serve as, as low-friction interfaces for water transport [35,36]. This chain-bridging behavior is further corroborated by simulations under zero external pressure difference. Under these conditions, the transport is driven purely by thermal fluctuations from the water reservoirs at both ends. This random thermal motion induces a high rate of bidirectional translocation, resulting in a stable total flow (Figure 8a) that remains essentially unchanged until the critical gap. However, as shown in Figure 8b, these forward and backward movements cancel each other out, leading to a net transmission efficiency η that is approximately zero across all gap sizes. This result explicitly clarifies that the high flow is due to chaotic thermal motion, not a net driving force. The fact that this flow (total motion) still decreases rapidly at the critical gap confirms the water chain’s breakdown and robustly underscores the intrinsic bridging capability.

**Figure 9:**
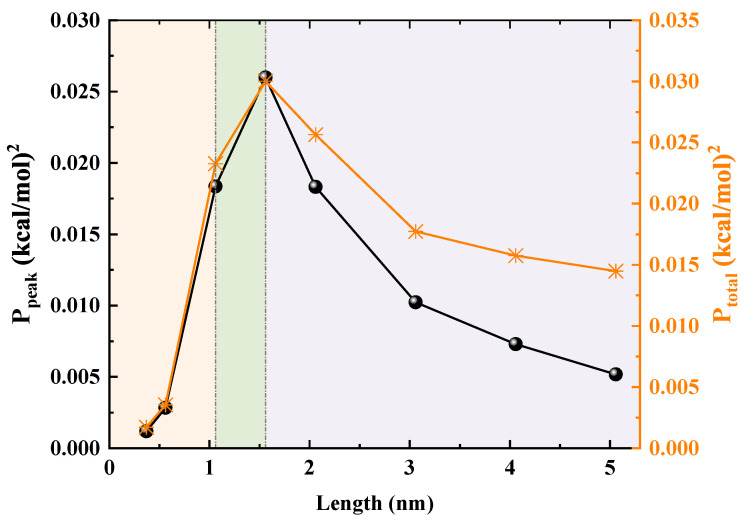
Peak frequency power ($P_{\mathrm{peak}}$) and total power ($P_{\mathrm{total}}$) obtained from the Fast Fourier Transform (FFT) analysis of the PMF curves as functions of tube length. The background colors distinguish the three distinct transport regimes: shallow well, ordered locking, and energy tunnel.

To transform the qualitative phenomenon into precise quantitative results, we apply FFT analysis to the PMF curves, as shown in Figure 9. This method extracts essential physical parameters from complex PMF profiles. In analyzing the spectral characteristics of the free-energy sequence $U(z_{n})$, we first remove the DC component by computing

(1) $$\tilde{U}\left(z_{n}\right)=U\left(z_{n}\right)-\frac{1}{N}\sum_{n=0}^{N-1}U\left(z_{n}\right),$$

yielding a zero-mean signal $\tilde{U}\left(z_{n}\right)$. We then apply the discrete Fourier transform

(2) $$F\left(k_{m}\right)=\Delta z\sum_{n=0}^{N-1}\tilde{U}\left(z_{n}\right)e^{-2\pi i\frac{mn}{N}},$$

where $k_{m}=\frac{m}{N\Delta z}$ and m=0,…,N−1, with frequency resolution Δk=1/(NΔz) and Nyquist frequency $k_{\mathrm{Nyquist}}=1/\left(2\Delta z\right)$. The two-sided power spectrum is $P_{2-\mathrm{sided}}\left(k_{m}\right)={\left|F\left(k_{m}\right)\right|}^{2}$, from which the single-sided power spectral density is constructed as

(3) $$P\left(k_{m}\right)=\left\{\begin{array}{cc} \frac{2}{N}{\left|F\left(k_{m}\right)\right|}^{2}, & k_{m}>0\\ 0, & k_{m}\leq 0 \end{array}\right..$$

We verify Parseval’s theorem via

(4) $$\sum_{m=1}^{N/2-1}P\left(k_{m}\right)\approx \sum_{n=0}^{N-1}{\tilde{U}}^{2}\left(z_{n}\right)\Delta z.$$

Finally, letting

(5) $$m_{\mathrm{peak}}=\arg\max_{1\leq m<N/2}P\left(k_{m}\right),$$

(6) $$k_{\mathrm{peak}}=\frac{m_{\mathrm{peak}}}{N\Delta z},$$

(7) $$\lambda_{\mathrm{peak}}=\frac{1}{k_{\mathrm{peak}}},$$

yields the index, frequency, and wavelength of the dominant component, and we characterize its strength and the total spectral energy by

(8) $$P_{\mathrm{peak}}=P\left(k_{\mathrm{peak}}\right)$$

and

(9) $$P_{\mathrm{total}}=\sum_{m=1}^{N/2-1}P\left(k_{m}\right).$$

We first identify the PMF’s primary wavelength ($\lambda_{\mathrm{peak}}$), which stabilizes at 2.5∼3.0 Å, closely matching the spacing between adjacent water molecules, thus providing a solid basis for further analysis. Next, the two key indicators are the peak frequency power ($P_{\mathrm{peak}}$), which measures the height of the main energy barriers in the PMF, and the total power ($P_{\mathrm{total}}$), which reflects the energy landscape’s overall roughness. In short SWCNTs with lengths below 1.06 nm, $P_{\mathrm{peak}}$ and $P_{\mathrm{total}}$ are low, indicating a flat, shallow-well PMF shape that lacks sufficient barriers to suppress thermal fluctuations at the tube openings. When the length reaches 1.06 nm, $P_{\mathrm{peak}}$ and $P_{\mathrm{total}}$ surge and peak at 1.56 nm, marking a transition to a rugged egg carton PMF profile. The elevated barriers effectively suppress backflow and thermal disturbances, thereby enhancing transport efficiency, though they also increase resistance to forward flow, leading to a noticeable inflection point in the flow. For lengths exceeding 1.56 nm, $P_{\mathrm{peak}}$ and $P_{\mathrm{total}}$ decline steadily, suggesting progressively lower energy barriers. Once these barriers fall below a certain threshold, the water chain’s ability to bridge gaps strengthens, enabling it to span larger structural gaps and significantly improving macroscopic stability.

**Figure 10:**
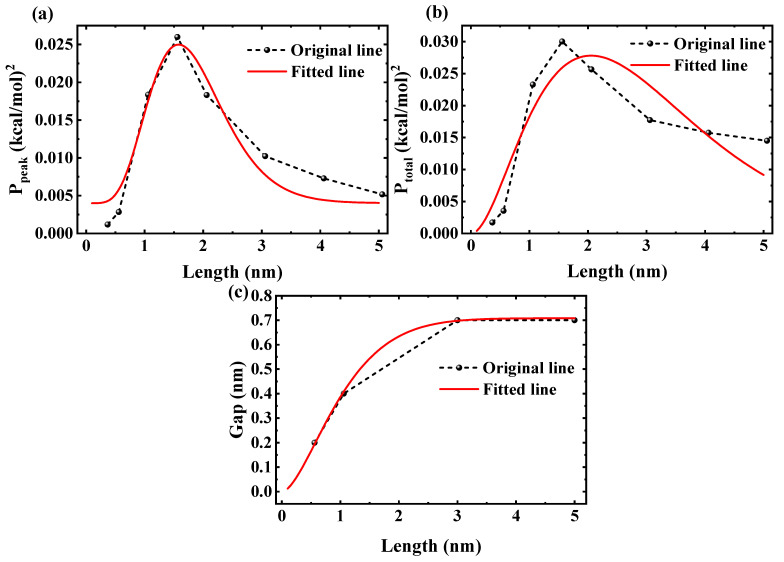
(**a**) Fit to the peak frequency power ($P_{\mathrm{peak}}$). (**b**) Fit to the total power ($P_{\mathrm{total}}$). (**c**) Critical gap distance for water-chain bridging versus nanotube length. In all three panels, the black line represents the original data, and the red line represents the fitted data.

To generalize the PMF evolution and length-driven trade-off observed in the FFT analysis, we propose a theoretical model treating the water chain as a flexible one-dimensional chain of *N* particles (N≈L/λ, λ≈0.275 nm) in an effective periodic potential [26,37]. We employ this model as a phenomenological description to link the static PMF landscape to dynamic transport kinetics. It is important to note that while the PMF is fundamentally a temperature-dependent free energy function (F(z,T)), the effective potential $V_{\mathrm{eff}}\left(z\right)$ is written here solely in terms of spatial coordinates *z*. In this approach, the critical parameter, the effective barrier height $E_{b}$, implicitly captures the effects of temperature as it is derived by fitting the PMF data calculated at T=300K

(10) $$V_{\mathrm{eff}}\left(z\right)=\frac{E_{b}}{2}\left(1-\cos\left(\frac{2\pi z}{\lambda }\right)\right)$$

governed by overdamped Langevin dynamics [38]

(11) $$\gamma \frac{dz_{i}}{dt}=-\frac{\partial V_{\mathrm{tot}}}{\partial z_{i}}+F_{\mathrm{ext}}+\sqrt{2\gamma kT}\;\eta_{i}\left(t\right)$$

where $V_{\mathrm{tot}}$ includes harmonic hydrogen bond interactions *k* approximately 5 to 10 kT/Å^2^. Here, the energy barrier $E_{b}\left(L\right)\propto \sqrt{P(L)}$ modulates the energy tunnel, with

(12) $$P\left(L\right)=aL^{b}e^{\left(-L/\xi \right)}+c,$$

fitted to the FFT data across all lengths. This form accurately captures the full curve. The power-law rise $L^{b}$ reflects critical buildup from shallow wells to ordered locking, akin to order parameter scaling in Ising-like models near criticality [39], peaking at L≈1.56 nm; the exponential decay $e^{\left(-L/\xi \right)}$ corresponds to low-damping tunnel formation in long tubes, with ξ as the correlation length matching confined fluid phase transitions [40,41]. Building on this foundation, the model further quantifies the chain-bridging phenomenon by considering the cumulative impact of ordering along the chain, which leads to an effective barrier form tailored for the critical gap

(13) $$\begin{array}{cc} d_{\mathrm{crit}}\left(L\right) & =\frac{kT\ln2\cdot \lambda }{E_{b}}\approx \kappa {\left(\int_{0}^{L}al^{b}e^{\left(-l/\xi \right)}\;dl\right)}^{1/4}, \end{array}$$

where κ≈1.65 is a scaling parameter in units consistent with $d_{\mathrm{crit}}$ in nm, fitted to MD trends. For this purpose, we refine $E_{b}\left(L\right)\propto {\left(\int_{0}^{L}al^{b}e^{\left(-l/\xi \right)}\;dl\right)}^{-1/4}$ to modulate the energy tunnel based on integrated strength. This refined approach accurately captures the monotonic increase and saturation. The cumulative integral represents the total ordering strength accumulated along the chain, building critically via the power-law rise and saturating after the exponential decay. Figure 10a,b show the squared peak and total PMF amplitudes, $P_{\mathrm{peak}}$ and $P_{\mathrm{total}}$, as functions of nanotube length *L*, with dashed lines from FFT data and solid lines representing fits via $P\left(L\right)=aL^{b}e^{-L/\xi }+c$ yielding $R^{2}\approx 0.925$ and 0.814, respectively. The power-law rise $L^{b}$ captures the critical ordering buildup and transition from shallow wells to locking, while the exponential decay $e^{-L/\xi }$ reflects damping in longer tubes, linking ξ to confined fluid phase transitions. Figure 10c presents the predicted critical gap distance $d_{\mathrm{crit}}\left(L\right)\approx \kappa {\left(\int_{0}^{L}al^{b}e^{-l/\xi }\;dl\right)}^{1/4}$ for chain-bridging, with κ≈1.65 fitted to MD trends and $E_{b}\left(L\right)\propto {\left(\int_{0}^{L}al^{b}e^{-l/\xi }\;dl\right)}^{-1/4}$. The integral accumulates total ordering strength, showing an increase from ∼0.2 nm at L≈0.56 nm to saturation at ∼0.7 nm for L≥3 nm.

**Figure 11:**
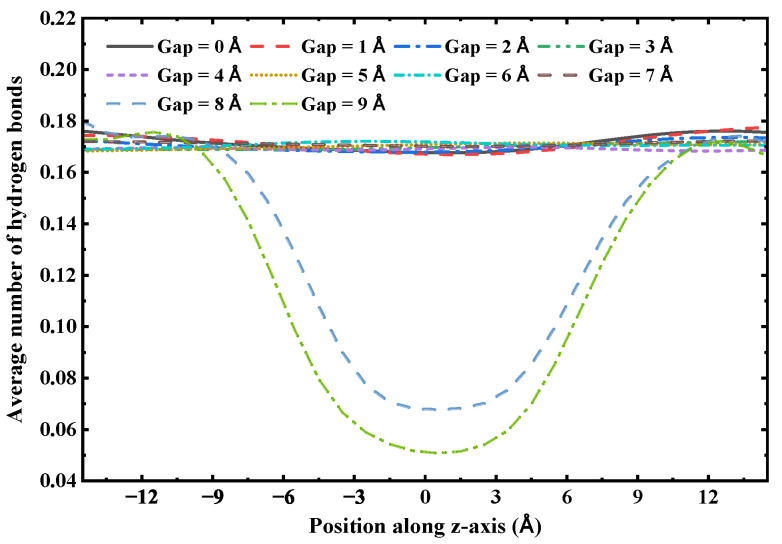
The axial distribution of the average number of hydrogen bonds in a 3 nm (6,6) SWCNT with varying gap sizes.

The axial distribution of hydrogen bonds indicates that the continuity of the hydrogen bond network is crucial for maintaining the structural integrity of the one-dimensional confined water chain, with its critical distance determined to be 7 Å. To illustrate this, we analyzed the distribution of the average number of hydrogen bonds along the tube axis in a 3 nm (6,6) SWCNT with varying gap sizes. As shown in Figure 11, when the gap size ranges from 0 to 7 Å, the hydrogen bond distribution remains consistent across the entire nanotube, including the gap region. However, when the gap size increases to 8 Å or 9 Å, the number of hydrogen bonds within the gap region decreases substantially. Since the structural stability of the single-file water chain within the (6,6) SWCNT is primarily determined by the intermolecular hydrogen bond network, this indicates that beyond a gap size of 7 Å, the water chain is unable to bridge the gap and maintain a continuous hydrogen bond network, resulting in a loss of structural integrity and subsequent rupture of the chain.

**Figure 12:**
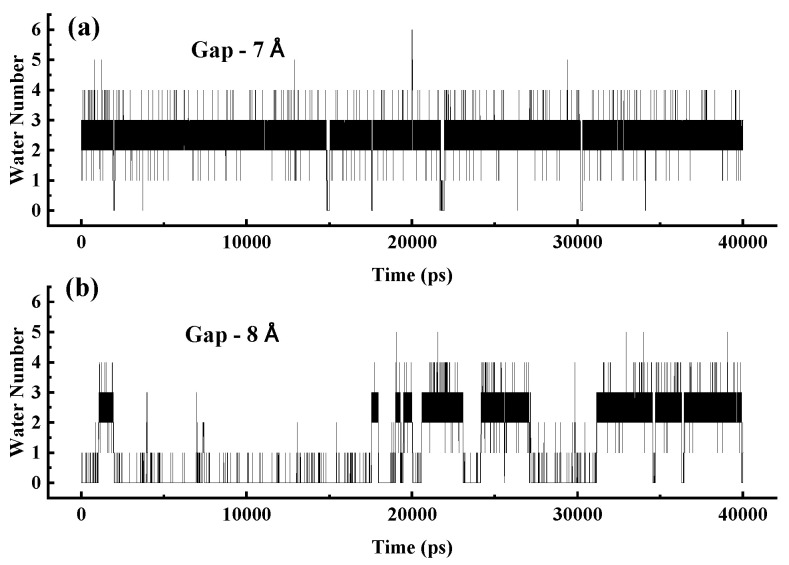
(**a**) Variation in the number of water molecules in a 7 Å gap within a 3 nm SWCNT over time. (**b**) Variation in the number of water molecules in an 8 Å gap within a 3 nm SWCNT over time.

When the gap is less than 8 Å, two water molecules can effectively bridge the gap, connecting the water chains on both sides. To demonstrate this stability, Figure 12 shows the variation in the number of water molecules within gaps in a 3 nm SWCNT over time. In panel (a), for a 7 Å gap, the number remains stable at 2–3, sufficient to establish a water bridge that restores the continuity of the broken water chain. In contrast, panel (b) shows that for an 8 Å gap, the number fluctuates significantly, often reaching zero, leading to the failure of the water-bridge mechanism and loss of structural and dynamic integrity in the water chain.

**Figure 13:**
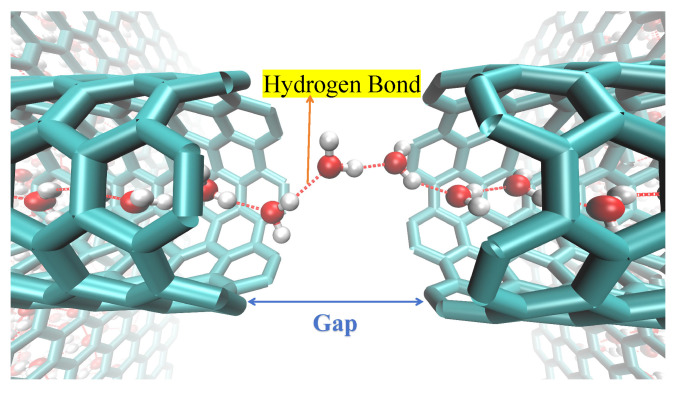
A snapshot capturing the movement trajectory. Under conditions of no external pressure, water molecules (red and white) can effectively bridge a gap of 7 Å inside a 3 nm long SWCNT, thereby restoring the continuity of the water chain (or bridging the water chain break) caused by this gap and forming a stable single-file water chain. Here, the cyan solid represents the nanochannel.

**Figure 14:**
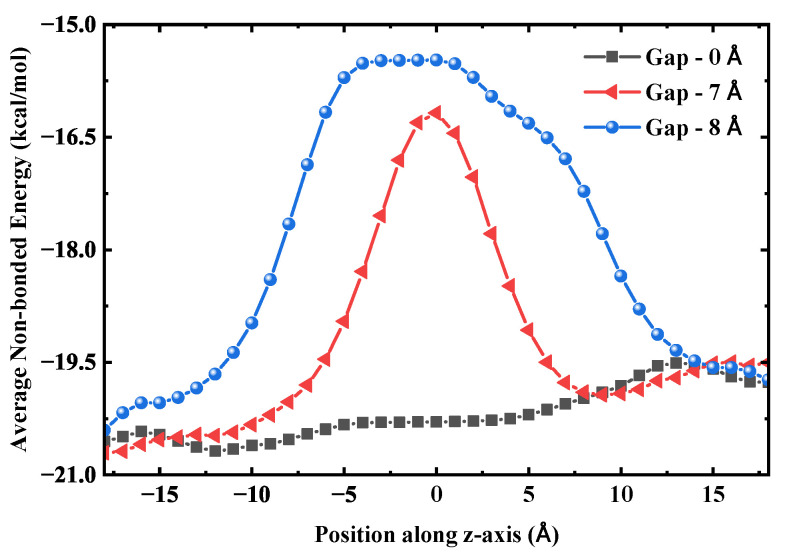
Average non-bonded interaction energy of water molecules with their surrounding environment along the axial direction, comparing an intact SWCNT, a fractured SWCNT with a 7 Å gap, and a fractured SWCNT with an 8 Å gap.

These molecules form a stable configuration and establish hydrogen bonds with the adjacent chains that, though stretched, remain sufficiently strong. This water-bridge mechanism, even in the absence of an external pressure difference, can effectively bridge breaks in the water chain, thereby endowing the water chain inside the tube with a strong ability to maintain its continuity. This mechanism is critically supported by the diffusive properties of water within the nanoscale bridge itself [42], as shown in Figure 13. This ensures the continuity of its structure, thereby maintaining a stable water flux. The hydrogen bond length is approximately 2.8 Å [43], and two molecules can span a distance of 5.6∼7.0 Å via stretched bonds. The 8 Å distance exceeds the limit, causing the hydrogen bonds to become overstretched and their strength to sharply decline, resulting in a high transport energy barrier. To examine this from an energetic perspective, we calculated the average non-bonded interaction energy of water molecules with their surrounding environment along the axial direction for three SWCNT configurations: an intact tube, a fractured tube with a 7 Å gap, and one with an 8 Å gap. As shown in Figure 14, the energy profile for the intact SWCNT is relatively flat, allowing smooth traversal without substantial barriers. For the 7 Å gap, a distinct energy barrier emerges. At 8 Å, both the height and width of this barrier increase, expanding the energetically unfavorable region and further hindering water transport, which correlates with the observed reduction in flux for larger gaps. This leads to a significant reduction in the average number of hydrogen bonds at the gap, signaling the rupture of the water chain and a substantial decrease in water flux.

**Figure 15:**
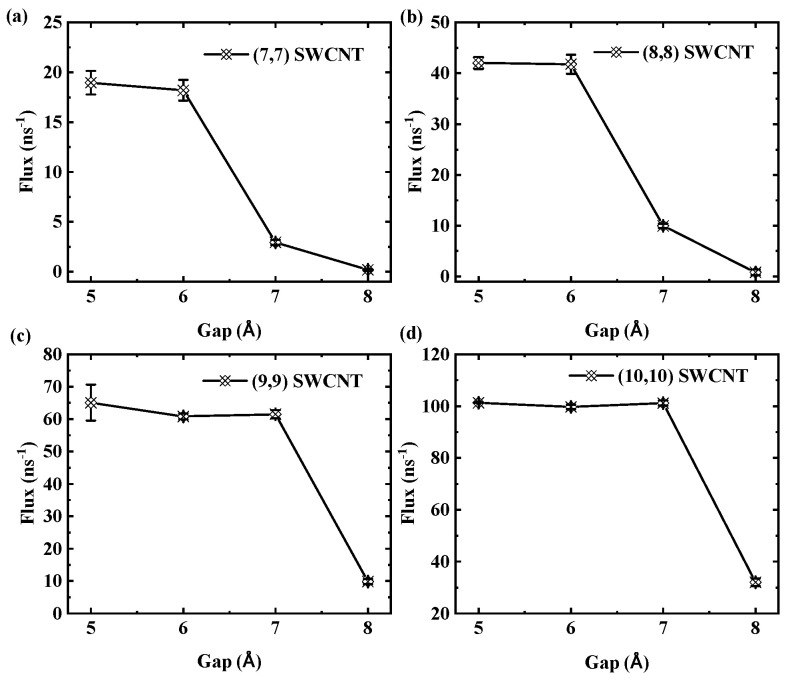
Flux as a function of gap size. (**a**,**b**) Flux for the (7,7) SWCNT and (8,8) SWCNT rapidly declines at a gap of 7 Å. (**c**,**d**) Flux for the (9,9) SWCNT and (10,10) SWCNT rapidly declines at a gap of 8 Å.

We further investigate the effects of tube diameter and thermodynamic parameters on the critical gap in SWCNTs. The results indicate a slight dependence of this value on diameter. Specifically, for 3 nm long tubes, the critical gap is 7 Å for (7,7) and (8,8) SWCNTs, while it is 8 Å for (9,9) and (10,10) SWCNTs. To illustrate this diameter dependence, Figure 15 shows the flux as a function of gap size for SWCNTs of different diameters with a fixed length of 3 nm. Panels (a) and (b) indicate that for (7,7) and (8,8) SWCNTs, the flux rapidly declines at a gap of 7 Å. In contrast, panels (c) and (d) show that for the larger-diameter (9,9) and (10,10) SWCNTs, the critical gap occurs at 8 Å, highlighting a subtle shift in transport thresholds with increasing diameter.

**Figure 16:**
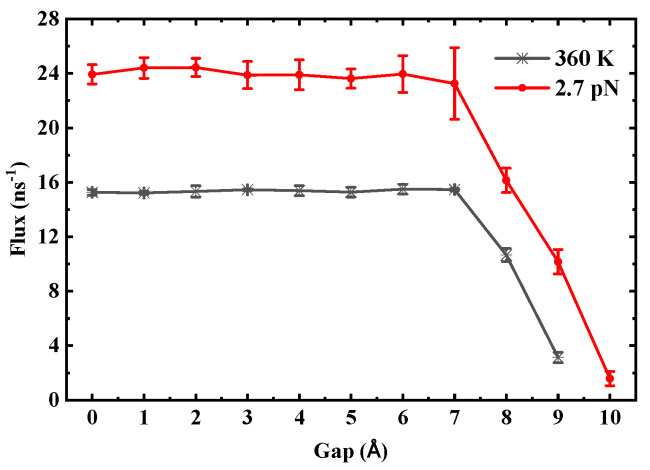
Flux as a function of gap in SWCNTs with a length of 3 nm, where the black line represents the condition with temperature increased to 360 K, and the red line represents the condition with a doubled driving pressure difference.

In contrast, the critical gap exhibits stability against thermodynamic perturbations. For (6,6) SWCNTs, even under conditions such as a temperature increase to 360 K or a doubled driving pressure difference, the critical gap remains consistently at 8 Å. To illustrate this robustness, Figure 16 shows the flux as a function of gap in SWCNTs with a length of 3 nm under varied conditions: temperature increased to 360 K (black line) and doubled driving pressure difference (red line). The critical gap remains stable at 8 Å in both cases, indicating minimal sensitivity to these external factors and underscoring the dominant role of nanotube geometry in determining transport thresholds.

Finally, we also considered the effect of the gap’s axial placement, as a defect is unlikely to be perfectly centered in a real-world system. Our preliminary investigations (from a related study) confirm that the water transport stability is indeed highly sensitive to the gap’s position. For a constant-length SWCNT (e.g., *L* = 5.06 nm), we found that moving the gap from the center towards either the entrance or the exit significantly reduces the critical gap at which transport fails. This observation is fully consistent with our PMF analysis (Figure 4), which shows high-energy barriers near the tube openings due to ‘end effects’. A gap located in these high-barrier regions compounds the total energy barrier for translocation, thus destabilizing the water chain’s continuity more easily than a centered gap.

## 4. Conclusions

This study confirms that the length of SWCNTs governs the fundamental trade-off between water transport rate, efficiency, and stability. As the length increases, the PMF transitions from a flat shallow well to a high-amplitude egg carton profile and ultimately to a low-resistance energy tunnel, a transport concept whose importance is increasingly recognized in accelerating nanoscale phenomena. These stages correspond to a shift in water transport modes that transitions from high-rate, low-efficiency, and poor-stability conditions in short tubes to low-rate, high-efficiency, and enhanced-stability conditions in longer tubes. Notably, the energy tunnel imparts an exceptional ability to maintain continuity to the water chain, enabling it to stably bridge a fracture gap of 7 Å. This finding provides a theoretical foundation for designing highly stable, resilient, and fault-tolerant nanodevices that can revolutionize applications in nanotechnology. This interpretation is robustly supported by our thermodynamic analysis (Figure 16), which demonstrates that the critical stability threshold is insensitive to a significant change in temperature (ΔT=60K). This strongly suggests that the stability threshold is dominated by the energetic/structural limitations of hydrogen bond integrity, with entropic contributions playing a secondary role in determining the critical fracture distance.

## Data Availability

The data presented in this study are available on request from the corresponding author.

## Supplementary Materials

The following supporting information can be downloaded at: https://www.mdpi.com/article/10.3390/molecules30234548/s1, Figure S1: Representative equilibration plot for total energy; Figure S2: Representative equilibration plot for system density; Figure S3: Representative equilibration plot for system temperature; Figure S4: Transport metrics (Flow, Flux, η) vs. length for the SPC/E model; Figure S5: Transport metrics (Flow, Flux, η) vs. length for the TIP4P/2005 model; Figure S6: Model sensitivity of critical gap; Figure S7: Instantaneous total Flow as a function of time for the short-tube regime (L=0.56 nm).

## Abbreviations

The following abbreviations are used in this manuscript:

FFT	Fast Fourier Transform
MD	Molecular Dynamics
NAMD	Nanoscale Molecular Dynamics
PMF	Potential of Mean Force
SWCNT	Single-Walled Carbon Nanotube
TIP3P	Transferable Intermolecular Potential with 3 Points
