# Supplementary material for: Tuning SWCNT Length to Optimize the Rate–Efficiency–Stability Triad in Nanofluidic Water Channels
Source: Molecules. 2025 Nov 25;30(23):4548. doi: 10.3390/molecules30234548 (PMC12692946; doi:10.3390/molecules30234548)
Supplement: Supplementary file 1 [file molecules-30-04548-s001.zip › molecules-3941319-supplementary.pdf]

Supplementary Information

Tuning SWCNT Length to Optimize the Rate-Efficiency-Stability Triad in Nanofluidic Water Channels

Shu-Peng Wang, Qi-Lin Zhang, Zhi-Jun Ma, Ju-Xiang Li, Zhen-Yan Lu and Rong-Yao Yang

S1. System Equilibration Validation

To demonstrate that all production runs started from a fully equilibrated state, representative equilibration plots are provided in Figures S1–S3. Before any data collection, all systems were rigorously equilibrated in the NPT ensemble (1 atm, 300 K) until key thermodynamic and structural properties reached a stable plateau with no systematic drift.

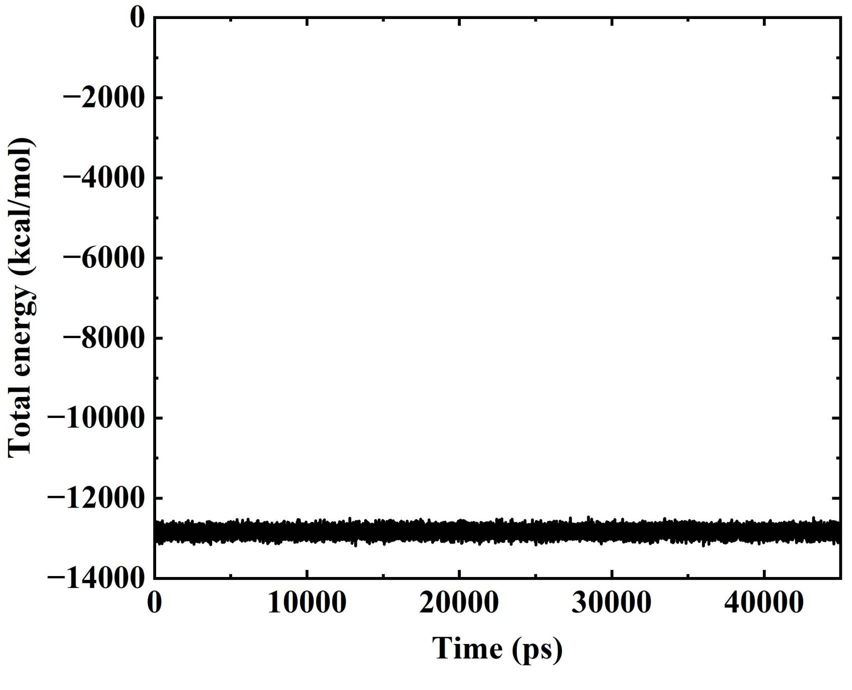

Figure S1. Representative equilibration plot for total energy. The total energy (kinetic+ potential) as a function of time, showing convergence to a stable plateau.

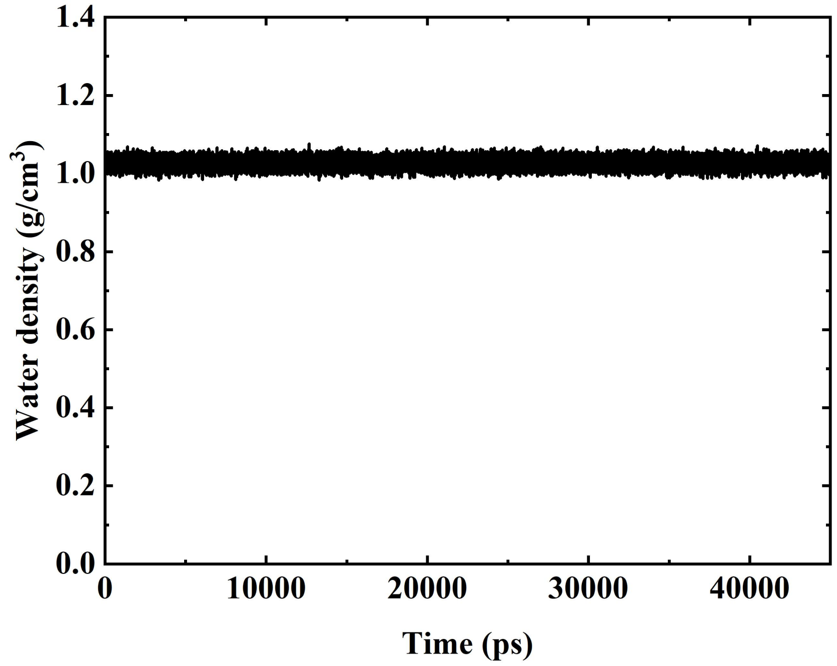

Figure S2. Representative equilibration plot for system density. The system density as a function of time, stabilizing around the target value of 1.0 g/cm³.

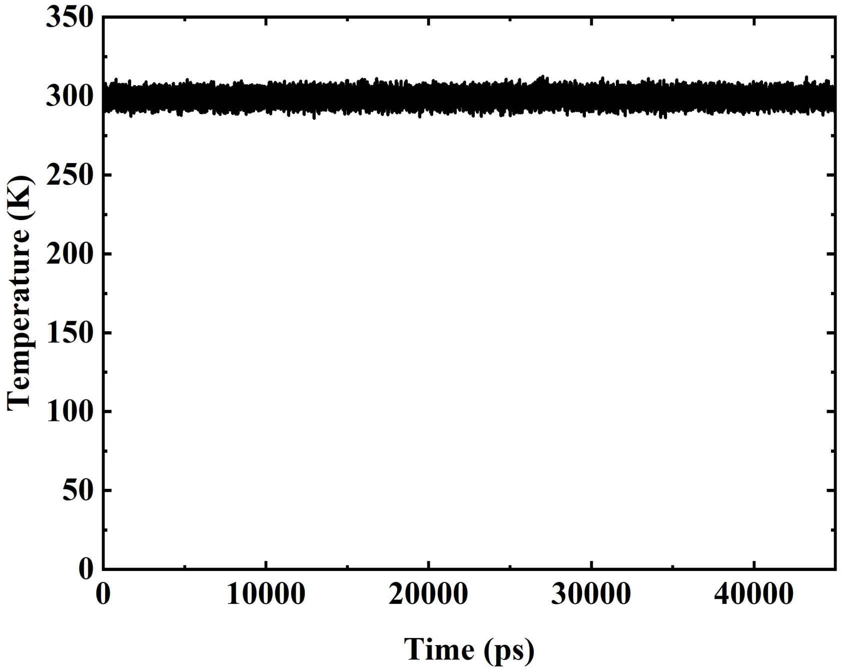

Figure S3. Representative equilibration plot for system temperature. The system temperature as a function of time, fluctuating stably around the target temperature of 300 K.

S2. Model Sensitivity Validation

To validate the robustness of the key physical phenomena presented in the main

text, we performed additional simulations using two other widely-adopted rigid water models: SPC/E and TIP4P/2005. All other simulation parameters were kept identical to those described in the main text. The results (Figures S4–S6) confirm that our primary conclusions are not artifacts of a specific force field choice.

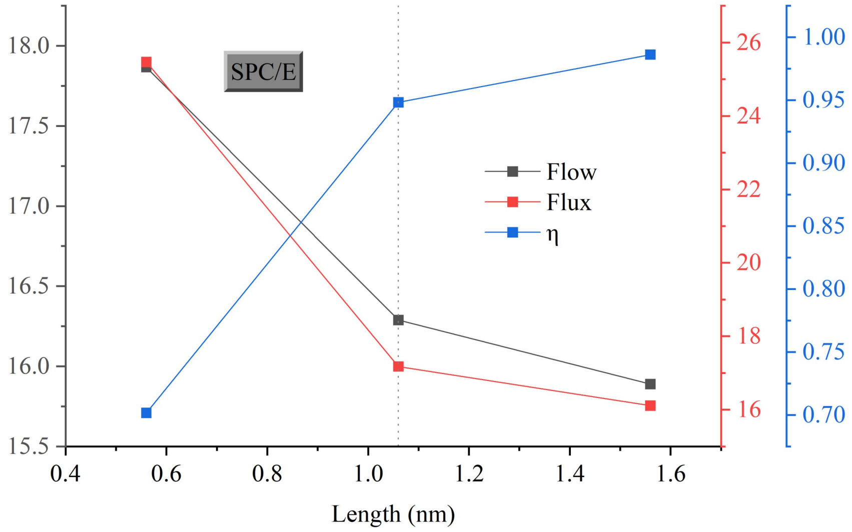

Figure S4. Transport metrics (Flow, Flux, *η*) vs. length for the SPC/E model. The plots for L = 0.56 nm, 1.06 nm, and 1.56 nm clearly show the same critical transition to high efficiency ($\eta \approx 1$) at L = 1.06 nm, confirming the robustness of this transition length.

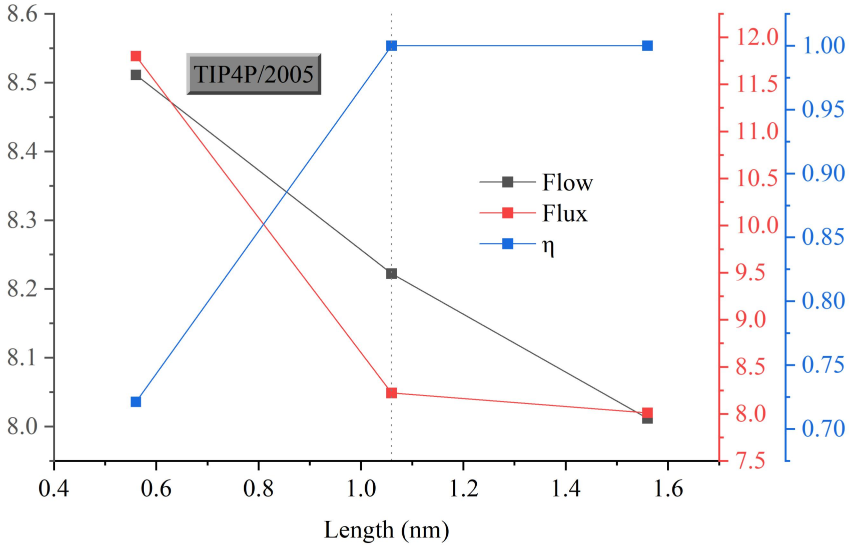

Figure S5. Transport metrics (Flow, Flux, *η*) vs. length for the TIP4P/2005 model. As with the SPC/E model, the TIP4P/2005 model also validates the critical transition to high efficiency at L = 1.06 nm.

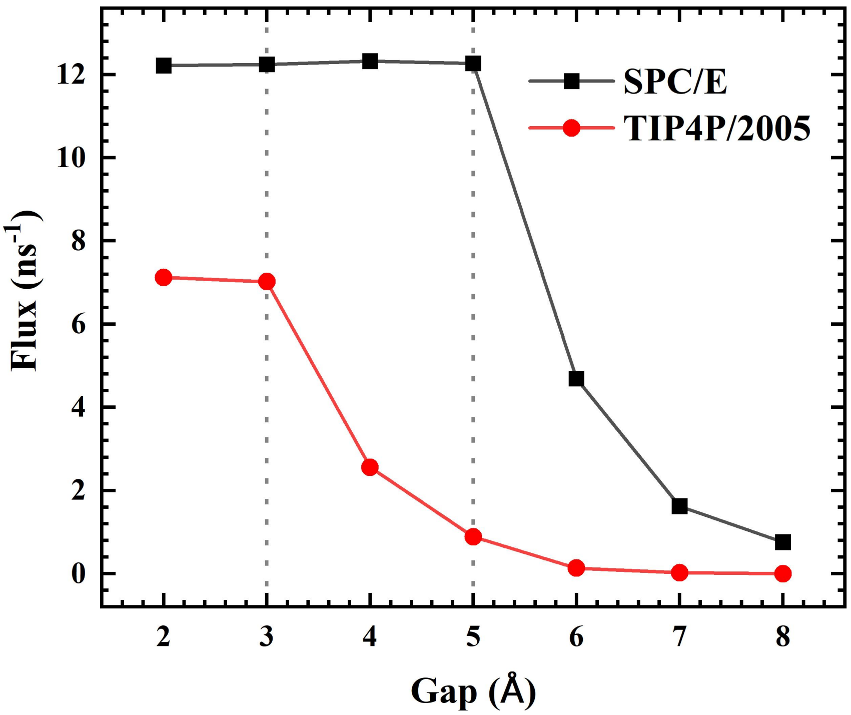

Figure S6. Model sensitivity of critical gap. Flux as a function of gap size for the SPC/E (black line) and TIP4P/2005 (red line) models. This confirms the existence of a critical gap in both cases, while also showing the expected quantitative dependence on the water model (critical gap ≈5 Å for SPC/E and ≈3 Å for TIP4P/2005).

S3. Time-Resolved Flow Validation for Short-Tube Regime

To provide direct quantitative evidence for the "burst-like" and chaotic conduction in short tubes (as discussed in the main text around Figure 5), we plot the instantaneous total Flow (total bidirectional translocation events) as a function of time for the representative L=0.56 nm system in Figure S7.

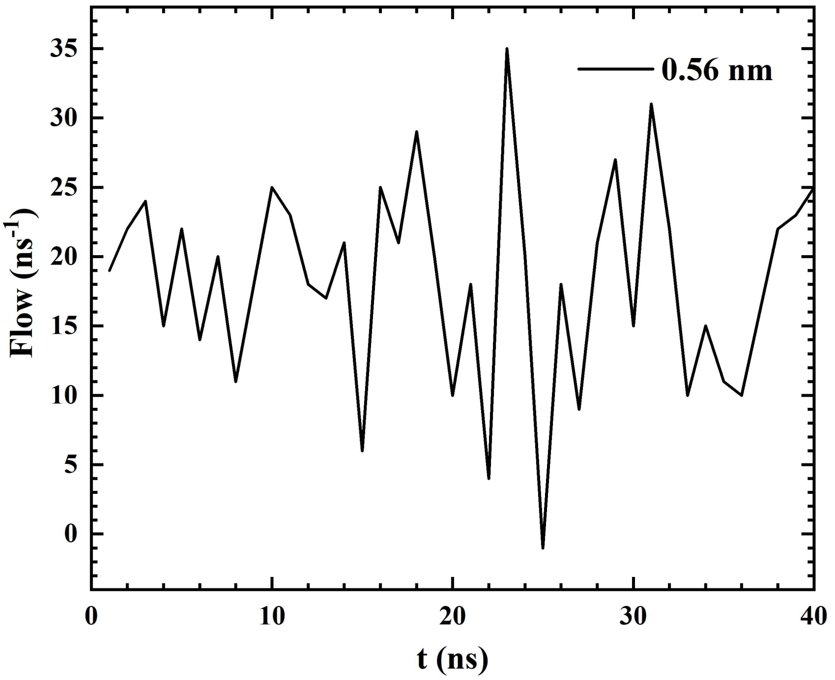

Figure S7. Instantaneous total Flow as a function of time for the short-tube regime (L=0.56 nm). The plot shows the total Flow (bidirectional translocations) is highly unstable and exhibits chaotic, burst-like behavior. The rapid and large-magnitude spikes in translocation events are direct evidence of the fluctuation-dominated transport, which results in a low net time-averaged Flux and low efficiency.
